# Getting out of energy-intensive and “dirty” transport for sustainable societies

**DOI:** 10.1016/j.xinn.2022.100339

**Published:** 2022-10-14

**Authors:** Becky P.Y. Loo, Kay W. Axhausen

**Affiliations:** 1 Department of Geography, University of Hong Kong, Hong Kong SAR 999077, China; 2 School of Geography and Environment, Jiangxi Normal University, Jiangxi 330022, China; 3 IVT, ETH Zürich, Zürich, Switzerland

## Introduction

Societies around the world have committed themselves to achieving the Paris goals of carbon dioxide (CO_2_) reduction and limiting global warming to 1.5°C with different levels of legal commitment. The transport sector is both a major use of energy and a major source of air pollutants, including CO_2_, nitrogen oxides, ozone, particular matter, and volatile organic compounds that cause climate change and harm human health.^1^^,^^2^ The recent Ukraine–Russia war triggered countries to evaluate a fossil–fuel-dependent transport system that is highly vulnerable. Achieving sustainable transport will have implications on all Sustainable Development Goals as good accessibility to food, health care, education, jobs, and resources are fundamental to meeting basic needs, supporting economic growth, and promoting equity.^3^

## The current transport policy dilemma

**Figure 1:**
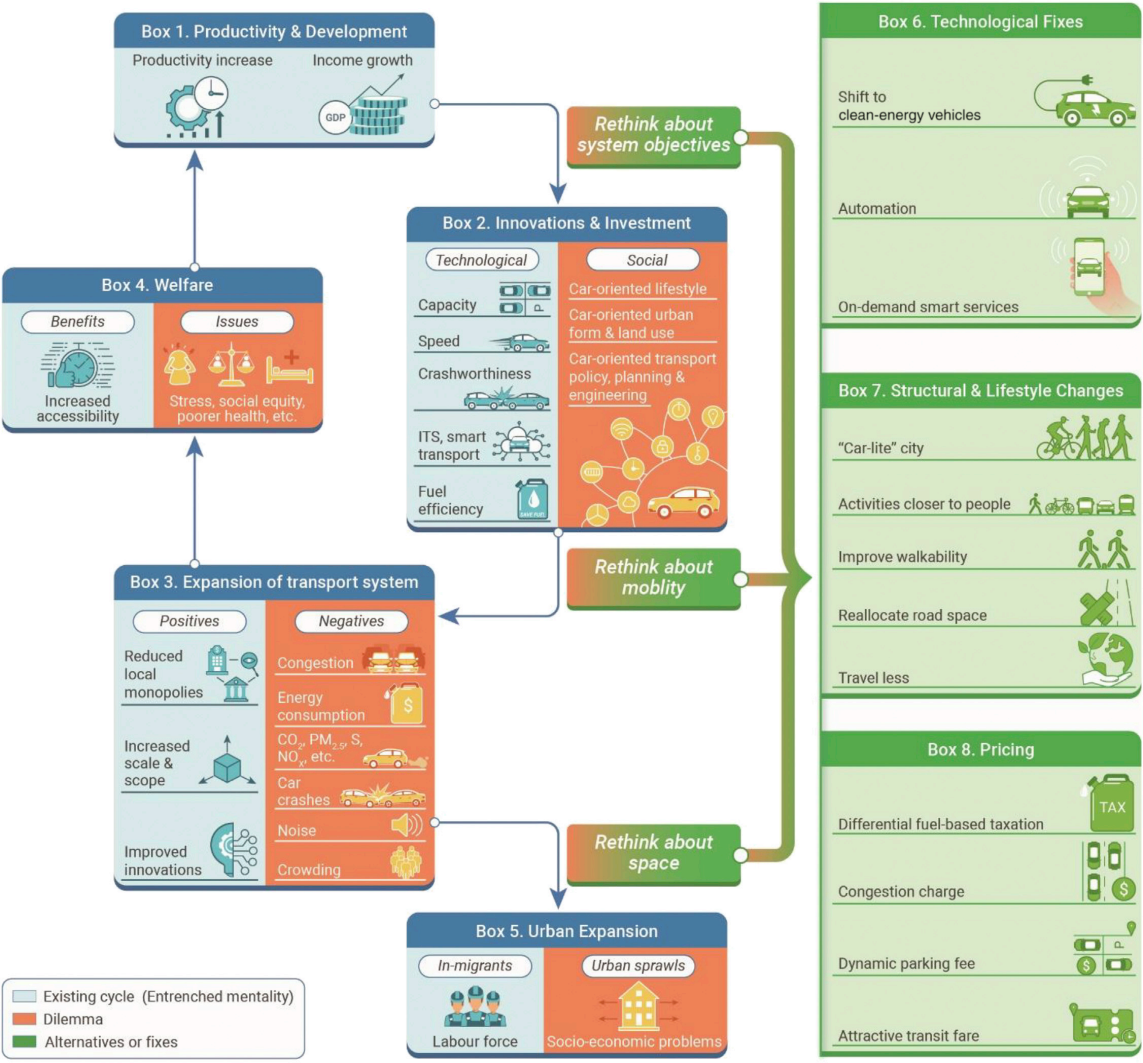
Pathways toward a green and energy-efficient transport system

Given the above urgent challenges, this commentary first sets out the current transport policy dilemma stopping the necessary changes toward sustainability and then discusses the alternatives. It hopes to help structure the current transport policy discussions and focus them on the necessary productive steps and design efforts to overcome the stasis of the current dilemma. Figure 1 conceptualizes the existing dilemma and possible ways of breaking away from the cyclical historical patterns of unsustainable transport entrenched in the existing transport policy mentality. With the primary goals of enhancing productivity and advancing development, societies have invested into accessibility in earnest (Figure 1, boxes 1 and 2). Transport policymakers intuitively understood that reducing the generalized costs of travel broke local monopolies, allowed increases in scale and scope, and supported innovation; in sum, it improved the welfare of the population and increased the tax base (Figure 1, box 4). As a result, the solutions have generally been capacity expansion of transport systems (Figure 1, box 3), that is, more, wider, and faster roads, more and faster rail lines, and more airports and faster/larger planes. Existing traffic congestion can be relieved for a while, but the induced demand effect of lower generalized costs of travel, in combination with population growth, overwhelms the system again. If the quality of transport is regionally different, the investment further attracts in-migration and urban expansion (Figure 1, box 5). The urban sprawl encourages the acquisition of more cars, increased vehicle mileage, and lower vehicle occupancy.

However, the failure to account for second order effects and negative externalities (“dilemma” in Figure 1) has led to unsustainable transport. Requiring developers of new buildings to provide on-site parking means that its cost is rolled over into prices of goods and services via rents or into property prices. Furthermore, car accessibility reduces the commitment to transit use and induces vehicle mileage, transport energy consumption, and greenhouse gas emissions. Nonetheless, increased economic wellbeing and rising expectations reduce the willingness to accept the negative externalities of traffic: noise, emissions, traffic crashes, congestion, and crowding within public transit. Further and large-scale transport capacity expansion, the preferred solution, is not easy anymore.

## A change of mentality

To break away from the existing unsustainable transport cycle requires a fundamental rethink at all levels because the existing transport dilemma has locked transport systems into a cycle of innovations and investment (Figure 1, box 2) that continually expands transport systems (Figure 1, box 3) and spurs urban expansion (Figure 1, box 4). These changes, in turn, perpetuate and aggravate the negative transport externalities associated with transport and urban expansion (Figure 1, dilemma in boxes 2–5) and lead to unsustainable societies and transport.

### Rethink about system objectives

As the ultimate goals that a system is set to achieve will implicitly determine its structure, mechanisms, and performances, a key change needed is to redefine the ultimate objective from productivity and development to comprehensive sustainability. Societies prefer policy solutions that change the technologies but not the structures of societies: technological fixes (Figure 1, box 6). Despite the business success of some transport network companies, large modal shifts toward more CO_2_-efficient modes are difficult. Electric vehicles and their charging infrastructure have only become more competitive recently. To speed up the market uptake, many countries will prohibit the sale of fossil fuel vehicles beginning 2025 or shortly afterward.^1^^,^^4^ Yet, there is the issue of the potential lack of clean electricity. Automated vehicles allow for the better use of existing infrastructure, increasing their capacity. The downside is the induced demand effect, which counter-balances the gains.

### Rethink about mobility

Secondly, we need to rethink about mobility, moving from a vehicle-oriented paradigm to the alternative of a people-oriented and place-based paradigm. The alternatives are to move beyond the expansion of transport systems and beyond car travel toward public transport, cycling, walking, and micro-mobilities and to move beyond physical travel toward virtual presence and accessibility. This approach aims to satisfy the activity needs of residents within walking distance (Figure 1, box 7). The most prominent idea is the “15-min city,” which incorporates ideas around “transit-oriented development,” “car-lite city,” or the “urban village.” Yet, it is unclear if it works for lower-density cities. The literature on urban hierarchy and human radii has, in addition, shown that different travel needs to maintain the social capital of our societies go beyond the radius of the 15-min city.^5^ Beyond the urban scene, longer-distance mobility is still needed to maintain social capital. The COVID-19 pandemic shows that some physical travel could be replaced by working from home, virtual meetings, and e-shopping. Various pop-up bike lanes have shown a pent-up demand for cycling and the feasibility of reallocating road space for a more radical change. It would be necessary to think through all the infrastructural adjustments necessary.

### Rethink about space

Last, but not least, we need to rethink space—effectively moving away from urban sprawl and urban expansion to more compact cities. To lock in the above changes, cities and regions require tools to manage demand. The fights about a reallocation of road space with fewer road-side parking spaces, fewer or narrower vehicle lanes, and more shared spaces are hard. Pricing has a major role to play (Figure 1, box 8). This refers both to the pricing of road space directly (e.g., congestion charging) and changing the relative cost or ease of using alternative less-energy-intensive transport modes. While welfare theory suggests pursuing first-best pricing, any socially accepted objective could be pursued. A spatially and temporally varying pricing mechanism allows for continuous adjustments of the price levels based on transport energy consumption or other proxy targets. The political discussion allows society to discuss the redistribution of revenues to address equity impacts.^3^ As sharing systems become more common, some road space can be freed up. The questions of weather dependency and associated needs for transport alternatives need to be solved.

## Conclusion and future directions

The preferred way out of the dilemma will vary by city and country, as their geographies, development, and laws make certain approaches easier to implement and easier to win the support. Technological fixes or pricing-based solutions are constrained by their associated rebound effects. Fundamental structural and lifestyle changes are also needed to achieve the transport energy transition. The 15-min city, with sustainable longer-distance transport modes like public transit and e-bikes, could become the vision for green mobility, which helps us to start thinking carefully about the objectives of urban transport policy and to find new starting points.
